# The Release of a Captive-Raised Female African Elephant (*Loxodonta africana*) in the Okavango Delta, Botswana

**DOI:** 10.3390/ani3020370

**Published:** 2013-04-29

**Authors:** Kate Evans, Randall J. Moore, Stephen Harris

**Affiliations:** 1 School of Biological Sciences, University of Bristol, Woodland Road, Bristol BS8 1UG, UK; E-Mail: s.harris@bristol.ac.uk; 2 Elephants For Africa, P.O. Box HA148 HAK, Maun, Botswana; 3 Elephant Back Safaris, Private Bag 332, Maun, Botswana; E-Mail: randalljaymoore@yahoo.fr

**Keywords:** African elephants, animal welfare, captive management, GPS, matrilineal groups, ranging behaviour, release to the wild, social behaviour

## Abstract

**Simple Summary:**

Managing captive elephants poses a significant challenge because of their complex social behaviour. While wild female elephants live in close-knit family groups of related individuals, captive herds often consist of unrelated animals. Some of the elephants in captive groups may be excluded by their companions and experience increased aggression, so that their welfare is compromised. There is no easy solution to this problem and novel approaches are required since slaughter of captive elephants is not publicly acceptable. We show that captive-raised female elephants can be released into the wild, survive and reproduce, and suggest that this management option should be explored further for female elephants currently held under various captive conditions.

**Abstract:**

Wild female elephants live in close-knit matrilineal groups and housing captive elephants in artificial social groupings can cause significant welfare issues for individuals not accepted by other group members. We document the release of a captive-raised female elephant used in the safari industry because of welfare and management problems. She was fitted with a satellite collar, and spatial and behavioural data were collected over a 17-month period to quantify her interactions with the wild population. She was then monitored infrequently for a further five-and-a-half years. We observed few signs of aggression towards her from the wild elephants with which she socialized. She used an area of comparable size to wild female elephants, and this continued to increase as she explored new areas. Although she did not fully integrate into a wild herd, she had three calves of her own, and formed a social unit with another female and her calf that were later released from the same captive herd. We recommend that release to the wild be considered as a management option for other captive female elephants.

## 1. Introduction

Wild female elephants form close long-lasting social bonds [1,2,3,4] and a female typically spends her entire life in her natal herd [4,5,6]. To reflect this, groups of captive elephants should ideally be based on related females and their offspring rather than disparate individuals brought together [7]. However, elephants are frequently exchanged between captive collections: these transfers are best documented for zoos. Of the female African elephants (*Loxodonta africana*) held in British zoos in 2005, only 1/15 of those ≥20 years old and 6/12 < 20 years old were living in the herd where they were born. Comparable figures for Asian elephants (*Elephas maximus*) were 0/24 ≥ 20 years old and 7/10 < 20 years old [8]. High rates of transfer of both adult and young female elephants continue between zoos and from the wild [9], and between other types of captive collections.

Transfers create a number of management problems for captive elephants. In zoos it reduces the survivorship of Asian elephants for four years post-transfer, and Asian elephant calves removed from their mothers when young tend to have poorer survival [10]. Tension and aggression between elephants will almost certainly be reduced in related groups that have grown up together [7]. While too many zoos appear to accept the fate of elephants that are not compatible with the rest of the herd [7], return to the wild is a potential management option for captive elephants. This has been done for a number of males used in the safari industry [11] and some from zoos [12,13]. Theoretically, this should be easier for males than females since adolescent males naturally disperse from their natal herd and integrate into bull society [14]. Fewer female elephants have been returned to the wild. Two zoo elephants from America were released into the Pilanesberg National Park, South Africa in 1982, where they ‘adopted’ orphaned calves introduced from the Kruger National Park, South Africa, and also had their own calves [12,15,16]. A female held in captivity in Kenya for 27 years was released into the Mkomazi Game Reserve in Tanzania in 1997, where she was observed with existing female herds and produced a male calf in November 2003 [17]. However, neither of these releases were fully documented, so we know little about how well captive-raised female elephants integrate into wild populations.

In this paper we describe the release of a female African elephant, Nandipa, orphaned as a one-year-old calf and reared as part of a herd of working elephants used in the safari industry. However, she was never fully accepted by the matriarch of the captive group, who would show extreme aggression towards her [18]. Possibly as a consequence, Nandipa became harder to manage, would not react to commands, and became aggressive. So it was felt that her welfare might be improved if she was released. This provided an opportunity to assess whether a captive-raised female elephant could be returned to the wild and integrate into a wild population. We collected behavioural and movement data to answer the following questions: (i) Is a female released by herself able to socialize with, and integrate into, a wild herd? (ii) Does a released female spend the majority of her time alone, with wild herds, or with known individuals? (iii) Does the area used by a released female reflect the area she knew prior to release or does she explore novel areas? (iv) Is the size of the area used by a released female comparable to the home ranges of female African elephants? (v) Is release to the wild a viable management option for captive-raised female elephants? 

## 2. Experimental Section

### 2.1. Nandipa’s History and Release

In 1989 Nandipa, along with five other youngsters orphaned by the cull in Kruger National Park, South Africa, were taken to the Okavango Delta, Botswana, where they joined three adult elephants (a 30-year old and 29-year old male and a 29-year old female) previously held in circuses and zoos in Canada and the United States. These formed the core of a working group of elephants, the Abu herd, that provided elephant-back safaris for tourists. The elephants were held at night in an electrified boma but chained separately. At first Nandipa and the other five youngsters accompanied the adults when they took tourists on safari. When seven years old, Nandipa was trained to wear a saddle to carry tourists.

Permission for the release was obtained from Botswana’s Department of Wildlife and National Parks, and the handling and release protocols were approved by the University of Bristol’s ethical review process. For a month prior to her release, Nandipa remained chained in the boma while the other elephants went out on safari and to graze. She was in auditory, but not tactile, communication with the rest of the herd during the night, and in infrasonic communication during the day. During this period she showed severe stereotypical behaviours (swaying and rocking for long periods), displacement behaviours such as trunk to mouth touches, and excessive secretions from her temporal glands.

Nandipa was fitted with a Globaltrack AWTSM2000E satellite collar (Africa Wildlife Tracking cc, Rietondale, Pretoria, South Africa) on 12 September 2003 without being sedated. The next day she was released from the boma (decimal degrees S19.41483, E22.58421) in the wildlife management area NG26 in the western part of the Okavango Delta [11] while the rest of the herd (2 adult males, 1 adult female, 3 adolescent females, 1 juvenile male and 1 female calf, where calves were 1–4 years, juveniles 5–9 years, adolescents 10–20 years, and adults ≥21 years old) were out. Nandipa was released into an area with which she was familiar and was regularly monitored until 10 February 2005 and sporadically thereafter. On 20 September 2010 she was sedated with etorphine hydrochloride (M99) by an experienced wildlife veterinarian, who darted her from a helicopter, the collar removed and the antagonist diprenorphine (M50/50) administered; she recovered within 10 minutes of being darted.

### 2.2. Data Collection

The VHF component of the satellite collar was used to locate Nandipa from the ground. Whenever she was seen (on average nine times per month), her social group (Table 1) and the sex, age and, where known, identification of any elephants she was with were recorded [14].

**Table 1:** Social groupings used in the analyses.

Code	Social status
**1**	Alone, no other elephant within 500 m
**2**	In a small group of bulls, 1–5 males within 500 m of each other
**3**	In a large group of bulls, >5 within 500 m of each other
**4 ^1^**	In a herd of male and female elephants within 500 m of each other
**5**	Within 500 m of the Abu herd

^1 ^While wild herds were predominantly females, they often contained young males that had yet to leave their natal herd and may have included mature bulls when a female was approaching or in oestrus.

**Table 2:** Activity codes used during the collection of observational and focal data.

Code	Activity	Description
**1**	Sleeping	Standing in one place with eyes closed for longer than one minute while not feeding
**2**	Feeding	Chewing or using the trunk to manipulate food items
**3**	Drinking	Intake of water
**4**	Social behaviours	Focal elephant physically interacting with at least one other elephant
**4.1**	Greeting	Raises trunk to mouth of another elephant
**4.1.1**		Another elephant greets focal elephant
**4.2/4.3**	Sparing/playing	Head to head contact and pushing between two or more elephants
**4.4**	Pushing from behind	Using tusks or resting trunk over back of the other elephant and pushing
**4.4.1**		Focal elephant is pushed from behind
**4.5**	Displaying	Destruction of vegetation without eating, crashing through vegetation, headshaking
**4.5.1**		Another elephant is displaying
**4.6**	Head over back	Standing or walking with head and/or trunk resting on back of another elephant
**4.6.1**		Another elephant with head and or trunk on back of the focal elephant
**5**	Mud bathing/dusting	Collection of dust or mud with trunk and then throwing it over themselves
**6**	Walking	Moving purposefully at a steady pace
**7**	Walking while feeding	Moving at a steady pace while chewing or manipulating food items
**8**	Standing	Standing in one place with eyes open for longer than one minute while not feeding
**9**	Vocalizing	
**9.1**		Vocalization by focal individual
**9.2**		Vocalization by known other
**10**	Running	Moving at pace, generally when alarmed
**11**	Other	Focal elephant does another activity e.g., pushes over tree to eat
**11.1**		Another elephant does another activity

Nandipa’s physical condition was scored on a 1–5 scale, where: 1 was emaciated; 2 very thin *i.e.*, the shoulder blades, pelvic bones and backbone were protruding; 3 normal *i.e.*, the shoulder blades, pelvic bones and backbone were noticeable; 4 good *i.e.*, there were slight depressions in front of the pelvic bones and shoulder blades, and the backbone was protruding slightly; and 5 fat *i.e.*, there was no sign of the shoulder blades, pelvic bones or backbone protruding and fat was hanging on the body [19]. Half scores e.g., 2.5 were used where it was uncertain which of two scores was most appropriate. Her behaviour was quantified during 30-minute focal observation periods: a GPS location (Garmin GPS III plus, Garmin International Inc, Olathe, KS, USA), the habitat she was in (see below), age, sex and distance to the nearest neighbour, and her behaviour (Table 2) were noted every five minutes. Any unusual behaviour outside the five-minute observation points was also noted.

Where possible, Nandipa was also tracked bimonthly from the air using a Piper J-3 Cub aeroplane (1946 Model, 100 HP) with H-aerials attached to each wing (African Wildlife Tracking cc) fixed at 45 degrees to the ground. The aerials were linked to a single Telonics R-4 receiver (Telonics Inc., Mesa, AZ, USA) through a switch box (African Wildlife Tracking cc). Once located, she was circled to collect data on her social grouping (Table 1), the number and sex of any other elephants present and distance from her nearest neighbour. Her lack of interest in the plane suggested that this procedure did not unduly disturb her or any elephants that were with her. Many small planes fly over the Okavango Delta, so the elephants may be habituated to them. Comparative data were collected from females in wild herds while driving set and random transects that sampled an area of 215 km^2^ centred on the release site [20]. Body condition scores and habitat type were recorded for any wild females seen during the 17 months when Nandipa was being monitored intensively. 

Habitat types from an existing map [21] were simplified to incorporate the main ecotypes. In the 215 km^2^ where wild females were sampled, grassland/floodplain constituted 51% of the area, other woodland (mainly *Acacia* spp., *Combretum* spp. and *Terminalia *forests) 41%, island vegetation (dominant species *Hyphaene petersiana* and *Phoenix reclinata*) 7%, and mopane (*Colophospermum mopane*) woodland 1% [20]. For the area covered by Nandipa, grassland/floodplain constituted 61%, other woodland 18%, island vegetation 2%, and mopane woodland 19%.

### 2.3. Other Elephant Releases

Three male elephants were released from the Abu herd shortly before Nandipa, a 14-year old (Mafunyane) on 1 February 2002, and a 16-year old (Thando) and a 9-year old (Seba) on 10 February 2003 [11,13]. All three were also orphans from culls in Kruger National Park and subsequently raised in the Abu herd; Mafunyane and Thando were two of the five youngsters moved to the Okavango Delta at the same time as Nandipa. Seba joined the herd in 1995, when about 18 months old. Subsequent to Nandipa’s release, a juvenile male (Pula), born in the Abu herd on 27 November 2000, was released on 16 May 2008 and Gika, Nandipa's companion while in the Abu herd, was released on 2 August 2011 with her 8-year-old female calf, Naya. Nandipa was familiar with all these elephants prior to her release.

### 2.4. Data Analysis

There were three distinct seasons: the rainy season (November–March), the flood season (April–September) and the dry season (October, sometimes into November). The sightings and behavioural data collected for Nandipa each season are summarised in Table 3. The total sightings data were used to analyze patterns of social grouping; the social focal data were used to analyze patterns of social behaviour within groups and nearest-neighbour distances; the focal data were used to analyze patterns of activity and the rate of vocalization; the individual satellite fixes were used to calculate home range sizes and patterns of habitat use; and consecutive 24-h downloads were used to calculate the daily distance travelled.

**Table 3:** Summary of the data collected on Nandipa in different seasons over the 17-month intensive monitoring period.

Data set	Rainy season	Flood season	Dry season	Total
Total sightings	58	68	29	155
Social focal data	17	21	5	43
Focal data	18	22	5	45
Individual satellite fixes	910	753	218	1,881
Consecutive 24-h downloads	268	195	60	523

Since the activity data were not normally distributed and could not be transformed, the effect of season and social grouping *i.e.*, whether Nandipa was with a wild herd or one or more released males, on the frequency of social behaviours and distance to nearest neighbour were analyzed separately using nonparametric statistics. Kruskal-Wallis tests were used for time budgets.

Area used was measured using Minimum Convex Polygons (MCPs) and kernels in ArcView 3.2; 70% kernels were used to define core areas. Data for daily distance travelled were log transformed and compared using one-way ANOVAs. The areas of available habitats were calculated using ArcView 3.2 based on an existing habitat map [21]. For Nandipa, patterns of habitat selection and use were quantified by comparing the composition of the area used *versus* the composition of the total available habitat using compositional analysis based on log ratio transformed proportions [22] and Compos 5.0 [23]. For wild females, the total available habitat was based on the 215 km^2^ study area. A lambda test (λ) was initially conducted to determine whether habitat use/selection deviated significantly from random. Where this was the case, habitats were ranked according to the degree of preference.

The underlying assumptions were met for all tests [24]. Analyses were conducted using MINITAB (version 14, 2004) and the results considered significant where α = 0.05.

## 3. Results

### 3.1. Social Behaviour

**Figure 1:**
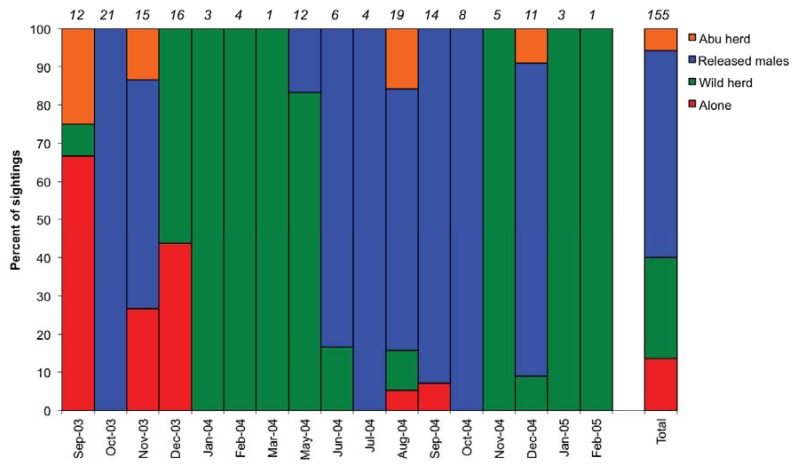
Percentage of sightings of Nandipa within different social groupings each month following her release. Numbers in italics show the sample size each month and the total sample size.

Immediately after her release, Nandipa spent a week alone in thick wooded vegetation, before starting to explore. After 17 days she joined up with all three of the previously released males. In December 2003 the three released males moved east away from the release site. Nandipa then briefly joined a wild herd and, when this herd moved away at the end of December 2003, she went with them. Over the next five months she was seen with wild herds 20 km southwest of the release site. In May 2004 the released males returned to the vicinity of the release site and Nandipa began to spend time with one or more of them and the wild males with which they associated. Thereafter Nandipa spent the majority of her time with one or more of the released males until February 2005, the end of the intensive monitoring period (Figure 1).

**Figure 2:**
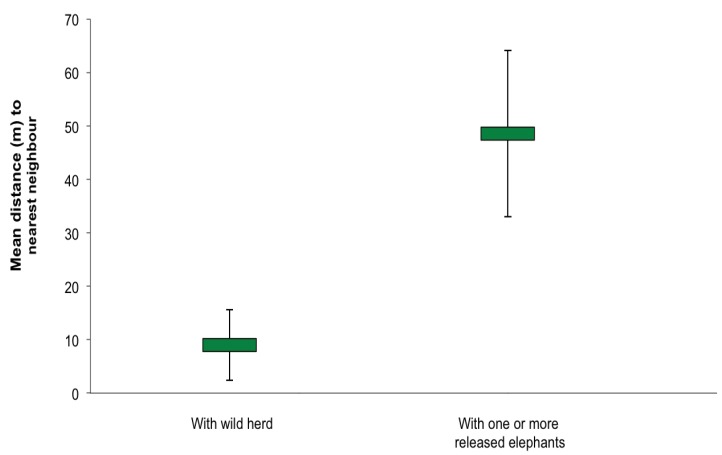
Distance to nearest neighbour when Nandipa was with wild herds and one or more of the released males. The figure shows means ± SE.

Over the 17 months she was monitored, Nandipa spent 55% of her time with one or more of the released males and the wild males with which they associated, and 28% of her time with wild herds. Twelve percent of her time was spent alone, while the remaining 5% was spent with the Abu herd. Distance to nearest neighbour was not affected by season (F_2,43_* = *1.100, *P *= 0.343) or time post-release (F_15,43_* = *1.610, *P *= 0.139). However, it was affected by social grouping (*t = *−3.56, *P *= 0.004), being significantly smaller when Nandipa was with a wild herd than when she was with the released males (Figure 2).

While there was a tendency for Nandipa to be more social (all social behaviours, greeting and sparring) and vocalize more (frequency per half hour focal) when she was with one or more of the released males than when she was with wild herds, the difference was not significant (Mann-Whitney *U:* all social behaviours, *W*_10,33_ = 203, *P *= 0.535; greeting, *W*_10,33_ = 221, *P *= 0.977; sparring, *W*_10,33_ = 231, *P *= 0.408; vocalization, Kruskal-Wallis: *H*_2_ = 2.45, *P *= 0.294) (Table 4).

Season did not affect the proportion of time Nandipa spent feeding (Kruskal-Wallis: *H*_2_ = 1.09, *P *= 0.579), walking (*H*_2_ = 2.11, *P *= 0.347) and sleeping *(H*_2_ = 4.33, *P *= 0.115), or the frequency of mud bathing/dusting (*H*_2_ = 0.39, *P *= 0.822) and other activities (*H*_2_ = 1.37, *P *= 0.504). Nor did her social grouping affect the proportion of time Nandipa spent feeding (*H*_2_ = 1.95, *P *= 0.376), walking (*H*_2_ = 2.35, *P *= 0.308) and sleeping (*H*_2_ = 0.41, *P *= 0.815), or the frequency of mud bathing/dusting *(H*_2_ = 0.45, *P *= 0.799) and other activities (*H*_2_ = 2.14, *P *= 0.342) (Table 4).

**Table 4:** Summary data (means ± SE) showing the frequency (all social interactions and vocalizations) and proportion of time (feeding, walking, sleeping, mudbathing/dusting and other activities) when Nandipa was with a wild herd (*N = *10) or one or more released males (*N = *33) and in the rainy (*N = *18), flood (*N = *22) and dry (*N = *5) seasons.

Activity	Wild herd	Released males	Rainy	Flood	Dry
All social interactions	0.20 ± 0.13	0.64 ± 0.27			
Greeting	0.10 ± 0.10	0.24 ± 0.18			
Sparring	0.10 ± 0.10	0.24 ± 0.24			
Vocalizations	0.00 ± 0.00	0.61 ± 0.28			
Feeding	52.9 ± 10.2	65.8 ± 4.9	58.8 ± 7.1	67.5 ± 6.4	62.8 ± 14.7
Walking	21.4 ± 5.7	13.0 ± 3.0	16.8 ± 4.3	15.0 ± 3.9	5.7 ± 5.7
Sleeping	12.9 ± 5.4	11.3 ± 4.1	13.5 ± 4.0	7.2 ± 4.6	25.7 ± 15.9
Mudbathing/dusting	2.9 ± 1.9	3.0 ± 1.2	3.4 ± 1.5	2.6 ± 1.5	2.9 ± 2.9
Other activities	1.4 ± 1.4	3.0 ± 1.6	0.8 ± 0.8	4.6 ± 2.4	0.0 ± 0.0

### 3.2. Home Range and Habitat Use

**Figure 3:**
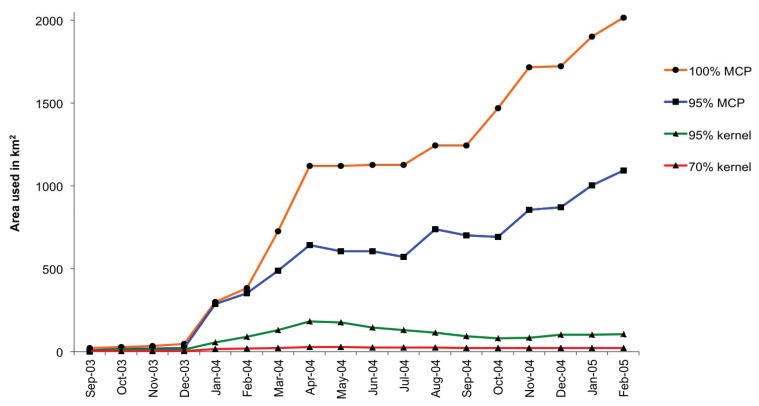
Cumulative changes in the area used by Nandipa in the months following her release. Area was measured using 100% and 95% minimum convex polygons (MCPs) and 95% and 70% kernels; 70% kernels were used to define core areas.

Between September 2003 and February 2005 Nandipa used an area of 2,017 km^2^ (100% MCP). Season did not affect the area used (Kruskal-Wallis 100% MCP: *H*_2_ = 0.31, *P *= 0.857; 95% MCP *H*_2_ = 0.56, *P *= 0.757; 95% kernel *H*_2 _= 4.67, *P *= 0.097; 70% kernel *H*_2_ = 5.19, *P *= 0.075), but the area used increased with time post-release (Figure 3), although her core area of activity remained fairly constant, with seasonal averages of 37.5 ± 11.2 (SE) km^2^. While Nandipa travelled up to 42 km from her release site, she continued to use the area in and around the release site following the period of intensive monitoring, and her core area was focused on the 20 km^2^ she used while part of the Abu herd. The daily distance travelled was affected by season (one-way ANOVA: *F *= 8.88, *P *< 0.001), with the mean daily distance travelled significantly lower in the dry (1.41 ± 0.14 km) than in the rainy (2.77 ± 0.18 km) or flood (2.61 ± 0.20 km) seasons.

Nandipa did not use the available habitats randomly (*χ*^2^_3_ = 10252, *P* < 0.001). While she used grassland/floodplain to the expected degree in all seasons, mopane woodland was avoided and island vegetation positively selected throughout the year. Other woodland was avoided during the flood and dry seasons but used to the expected degree during the rainy season; this differed from wild females living in the same area [13]. Nandipa's body condition scores were comparable to wild female elephants during the rainy and flood seasons but lower in the dry season (Table 5).

**Table 5:** Number of body condition scores recorded for Nandipa each season (left figures) and for wild females (right figures) seen during the same period that Nandipa was intensively monitored.

Condition score ^1,2^	Rainy season	Flood season	Dry season
1	0 0	0 0	0 0
2	0 0	0 0	1 0
2.5	5 2	1 0	2 0
3	2 6	4 0	2 3
3.5	8 1	2 0	0 1
4	4 9	11 7	0 4
4.5	0 1	0 1	0 1
5	0 0	0 0	0 0
**Total**	**19 19**	**18 8**	**5 9**

^1^ 1 denotes emaciated, 2 very thin, 3 normal, 4 good and 5 fat; see Experimental Section for details [19]. ^2^ There was a minimum period of a week between recording body condition scores for Nandipa.

### 3.3. Other Welfare Indicators

The excessive secretions from Nandipa’s temporal glands ceased on release, as did her excessive swaying and displacement behaviours. She gave birth to a male calf in July 2006, 34 months after release, and another male calf in December 2009. A third calf was born in April 2013; its sex had not been established at the time of publication.

Following his release on 16 May 2008, Pula spent the majority of the next two years with Nandipa before becoming independent; he returned occasionally until 16 September 2011, when he was shot 19 km away as a problem animal. After their release on 2 August 2011, Gika and her 8-year-old female calf, Naya, joined Nandipa and subsequently spent the majority of their time with Nandipa and her two calves. Nandipa appeared to be making most decisions on movements and activities and these five elephants appeared to be functioning as a cohesive group. 

## 4. Discussion

We used several measures to determine whether Nandipa’s release could be judged successful. Her ability to integrate into cow society was of particular concern, since female society consists of closed matrilineal groups, and she was released because of conflict with the matriarch in the Abu herd. The second key issue was whether Nandipa would explore her surroundings and exploit different habitats and food sources, and whether she would be able to maintain good body condition throughout the year. A third, and standard, measure of success was whether she survived long enough to breed [25]. While interactions with humans can be a concern with captive-raised animals, Nandipa showed no interest in humans or aggression towards them following her release, and so we do not consider this issue further.

### 4.1. Social Integration

While rare, lone female African elephants have been observed in the wild [18,26]. A female with no sisters whose mother has died may become peripheral to the main herd, moving more frequently with just her own offspring [6], and a female and her young will forage on their own when food availability drops below the levels needed to sustain large herds [27]. Rarely, wild unrelated females may associate with each other and unrelated family groups may fuse [1,5], and elephants from culled or introduced populations will form new ‘families’ made up of unrelated individuals [16,28]. Escaped tame female Asian elephants in India have joined wild herds [29], although these may have been related since, at that time, captive elephants were wild caught when young. 

Similar problems with integration have been reported with other species in which females form close bonds. A hand-reared female meerkat (*Suricata suricatta*), for instance, was not socially accepted into a group of wild meerkats when released, although she did mate and start her own group [18]. However, problems with integration are not invariably the case in species with matrilineal groups [30]. In chimpanzees (*Pan troglodytes*), where males form patrilineal groups and females disperse, released females integrated into wild groups for extended periods but were never fully accepted, whereas encounters with wild chimpanzees were a major cause of mortality for the released males [31].

Despite being released alone and not having met or socialized with any of the wild herds in the area, Nandipa spent over a quarter of her time with wild herds and visited new areas with them during the 17 months she was monitored intensively. When with wild herds, Nandipa was significantly closer to other elephants than when she was with one or more of the released males, although she was still generally on the periphery of groups. Her nearest neighbour distances were greater than the median of 1–3 metres for wild males of all ages [14] and 5 metres for captive-released males [11] in the same area. Nandipa also tended to greet more when in wild herds, suggesting she was socializing with them: females tend to be more tactile than males [32]. Although Nandipa was not fully accepted by, or integrated into, a wild herd, there were few signs of aggression towards her by members of the wild herds. We only recorded one incidence of sparring when she was with a wild herd, and that was with an adolescent male. It is usual for adult female elephants to spar, and Nandipa mainly sparred when she was with one or more of the released males.

However, Nandipa was mainly with wild herds during the rainy season when resources were not limited and elephants were able to spend more time socializing and playing [5]. In other populations, large aggregations of elephants are sighted during the rainy season, and families may join into bond groups and clans [2,5]. Thus the wild herds may have been more willing to accept her when competition for resources was low. Also, the area where our study took place was dominated by bulls, and most wild herds generally moved out of the area after a day or two. It is likely that these were non-dominant herds using peripheral areas [20]; whether this influenced Nandipa’s social interactions with them is unknown.

The majority of the wild elephants that associated with Nandipa were young males 8 to 12 years old, and she spent over half her time with one or more of the released males, possibly suggesting that the enduring social bonds with other members of their natal group [4,33] extend to males. Adult females do not normally associate with males other than adolescent herd members prior to independence or during mating; Nandipa’s social interactions with the released males highlight the importance of “knowing” individuals in elephant society.

### 4.2. Spatial and Habitat Use

The area used by Nandipa was larger than that recorded for most populations [34,35,36,37,38,39,40,41,42,43]. At first she continually explored new areas, while her core area remained fairly constant, although she explored less after the period of intensive monitoring and the area she used stabilised. Despite the large area explored, Nandipa travelled less far each day than female elephants elsewhere in Africa [41]. While seasonal changes in the size of the area she used were not significantly different, Nandipa tended to use a smaller area during the dry season. This may be because she was returning to familiar areas to locate limited resources, or it may be an artefact of the small sample size for this short period: female elephants generally have different wet and dry season ranges [44].

Nandipa’s habitat use differed from that of wild female elephants in the same area in that she avoided mopane woodland, whereas wild female elephants used this habitat more than expected. Island vegetation was positively selected while grassland/floodplain was used to the expected degree. However, of necessity we compared satellite collar data from Nandipa with observational data on wild herds, since they were not resident in the area and none were collared. So these differences may reflect differences in methods of data collection. Alternatively, prior experience may have influenced her decisions as the Abu herd rarely utilized mopane woodland.

More importantly, in the 17 months following release Nandipa was able to maintain body condition scores similar to those of wild females during the rainy and flood seasons, although she was in poorer condition during the short dry season. This may reflect her lack of familiarity with the extended area she explored post release; wild herds would have known the best foraging areas when resources were limited. The low body condition scores for the dry season may also be influenced by the fact that her release occurred immediately before the first of the two dry seasons she was intensively monitored, and she had lost condition while held in the boma prior to release. Ideally, she would have been released during a period of maximum food availability, but this was not possible due to the rapid decline in her condition and welfare. Following her release, Nandipa’s body condition scores improved over time, suggesting a decrease in stress and an improvement in her welfare.

### 4.3. Other Welfare Indicators

Wild female elephants can give birth when 9-years old [45], although they normally have their first calf at 11 to 14 years [46,47]. Nandipa was released when fifteen years old: she had not bred, although she had opportunities to breed with both wild and captive males when part of the captive herd and mounting had occurred. Whether this reproductive failure was related to her status within the Abu herd is unknown. Since gestation is around 22 months, Nandipa mated successfully for the first time approximately a year after release. Her first two calves were thriving in April 2013, when her third calf was born. 

## 5. Conclusions

It is important to consider the long-term welfare of captive elephants, especially when they consist of groups of unrelated individuals. It is to be expected that some of these elephants, as was the case for Nandipa, will not be accepted by their companions and their welfare compromised. While this was a case study, and so limited by sample size, it shows that captive-raised females can be released into the wild, survive, reproduce and spend time with, and move with, wild herds. However, it also highlighted the importance for released elephants to be able to socialize with known individuals and of social bonding at an early age in female elephants. There may be greater welfare concerns when female elephants are released by themselves; our data suggest that future releases of captive female elephants should include one or more other known individuals. In addition, each individual case should be carefully considered to ensure that the welfare of the individual is not further compromised, the area is suitable for the release, and that humans are not put at risk. This study, and that on male elephants [11], show that releasing wild-born, captive-raised elephants is a valid long-term management strategy and should be explored further using elephants from different types of captive collection.

## References

[1] Charif R.A., Ramey R.R., Langbauer W.R., Payne K.B., Martin R.B., Brown L.M. (2005). Spatial relationships and matrilineal kinship in African savanna elephant (*Loxodonta africana*) clans. Behav. Ecol. Sociobiol..

[2] Wittemyer G., Douglas-Hamilton I., Getz W.M. (2005). The socioecology of elephants: Analysis of the processes creating multitiered social structures. Anim. Behav..

[3] Archie E.A., Moss C.J., Alberts S.C. (2006). The ties that bind: genetic relatedness predicts the fission and fusion of social groups in wild African elephants. Proc. Roy. Soc. Lond. B.

[4] Archie E.A., Moss C.J., Alberts S.C., Moss C.J., Croze H., Lee P.C. (2011). Friends and relations: Kinship and the nature of female elephant social relationships. The Amboseli Elephants: A Long-Term Perspective on a Long-Lived Mammal.

[5] Moss C. (1988). Elephant Memories: Thirteen Years in the Life of an Elephant Family.

[6] Moss C.J., Lee P.C., Moss C.J., Croze H., Lee P.C. (2011). Female social dynamics: Fidelity and flexibility. The Amboseli Elephants: A Long-Term Perspective on a Long-Lived Mammal.

[7] Veasey J. (2006). Concepts in the care and welfare of captive elephants. Int. Zoo Yearbook.

[8] Harris M., Sherwin C., Harris S. (2008). The Welfare, Housing and Husbandry of Elephants in UK Zoos: Final Report. http://randd.defra.gov.uk/Default.aspx?Menu=Menu&Module=More&Location=None&Completed=1&ProjectID=13192.

[9] Anon (2012). Elephants Transferred. http://www.elephant.se/elephant_transfers.php?year=2012.

[10] Clubb R., Rowcliffe M., Lee P., Mar K.U., Moss C., Mason G.J. (2008). Compromised survivorship in zoo elephants. Science.

[11] Evans K., Moore R., Harris S. (2013). The social and ecological integration of captive-raised adolescent male African elephants (*Loxodonta africana*) into a wild population. PLoS One.

[12] Moore R.J., Munnion C. (1989). Back to Africa.

[13] Evans K.E. (2006). The Behavioural Ecology and Movements of Adolescent Male African Elephant (*Loxodonta africana*) in the Okavango Delta, BOTSWANA. Ph.D. Thesis.

[14] Evans K.E., Harris S. (2008). Adolescence in male African elephants, *Loxodonta africana*, and the importance of sociality. Anim. Behav..

[15] Anderson J.L. (1986). Restoring a wilderness: The reintroduction of wildlife to an African national Park. Int. Zoo Yearbook.

[16] Moore R.J. (2000). Elephants for Africa.

[17] Anon Individual stories: Nina. http://www.bornfree.org.uk/uploads/media/board_13.pdf.

[18] Evans K.E. (1995).

[19] Poole J.H. (1982). Musth and Male-Male Competition in the African Elephant. Ph.D. Thesis.

[20] Evans K.E., Harris S. (2012). Sex differences in habitat use by African elephants (*Loxodonta africana*) in the Okavango Delta, Botswana: Is size really the deciding factor?. Afr. J. Ecol..

[21] Jellema A., Ringrose S., Matheson W. (2002). Vegetation Mapping in Northern Botswana.

[22] Aebischer N.J., Robertson P.A., Priede I.G., Swift S.M. (1992). Practical aspects of compositional analysis as applied to pheasant habitat utilization. Wildlife Telemetry: Remote Monitoring and Tracking of Animals.

[23] Smith P.G. (2003). Compos Analysis Version 5.0 User’s Guide. Smith Ecology Microsoft® Excel Tool for Compositional Analysis.

[24] Zar J.H. (1984). Biostatistical Analysis.

[25] Sarrazin F., Barbault R. (1996). Reintroduction: Challenges and lessons for basic ecology. Trends Ecol. Evol..

[26] Mubalama L. (2000). Population and distribution of elephants (*Loxodonta africana africana*) in the central sector of the Virunga National Park, eastern DRC. Pachyderm.

[27] Mutinda H., Poole J.H., Moss C.J., Moss C.J., Croze H., Lee P.C. (2011). Decision making and leadership in using the ecosystem. The Amboseli Elephants: A Long-Term Perspective on a Long-Lived Mammal.

[28] Slotow R., Garai M.E., Reilly B., Page B., Carr R.D. (2005). Population dynamics of elephants re-introduced to small fenced reserves in South Africa. S. Afr. J. Wildl. Res..

[29] Sanderson I.T. (1963). The Dynasty of Abu: A History and Natural History of the Elephants and Their Relatives Past and Present.

[30] Chepko-Sade B.D., Sade D.S. (1979). Patterns of group splitting within matrilineal kinship groups. Behav. Ecol. Sociobiol..

[31] Goossens B., Setchell J.M., Tchidongo E., Dilambaka E., Vidal C., Ancrenaz M., Jamart A. (2005). Survival, interactions with conspecifics and reproduction in 37 chimpanzees released into the wild. Biol. Conserv..

[32] Poole J.H., Granli P., Moss C.J., Croze H., Lee P.C. (2011). Signals, gestures, and behavior of African elephants. The Amboseli Elephants: A Long-Term Perspective on a Long-Lived Mammal.

[33] Pinter-Wollman N., Isbell L.A., Hart L.A. (2009). The relationship between social behaviour and habitat familiarity in African elephants (*Loxodonta africana*). Proc. Roy. Soc. B..

[34] Western D., Lindsay W.K. (1984). Seasonal herd dynamics of a savanna elephant population. Afr. J. Ecol..

[35] Dunham K.M. (1986). Movements of elephant cows in the unflooded Middle Zambezi Valley, Zimbabwe. Afr. J. Ecol..

[36] Viljoen P.J. (1989). Spatial distribution and movements of elephants (*Loxodonta africana*) in the northern Namib Desert region of the Kaokoveld, South West Africa/Namibia. J. Zool..

[37] Lindeque M., Lindeque P.M. (1991). Satellite tracking of elephants in northwestern Namibia. Afr. J. Ecol..

[38] Tchamba M.N., Bauer H., De Iongh H.H. (1995). Application of VHF-radio and satellite telemetry techniques on elephants in northern Cameroon. Afr. J. Ecol..

[39] Thouless C., Kangwana K. (1996). Satellite tracking of elephants. Studying Elephants.

[40] De Villiers P.A., Kok O.B. (1997). Home range, association and related aspects of elephants in the eastern Transvaal Lowveld. Afr. J. Ecol..

[41] Blake S., Douglas-Hamilton I., Karesh W.B. (2001). GPS telemetry of forest elephants in Central Africa: results of a preliminary study. Afr. J. Ecol..

[42] Osborn F.V. (2004). The concept of home range in relation to elephants in Africa. Pachyderm.

[43] Galanti V., Preatoni D., Martinoli A., Wauters L.A., Tosi G. (2006). Space and habitat use of the African elephant in the Tarangire-Manyara ecosystem, Tanzania: implications for conservation. Mamm. Biol..

[44] Grainger M., van Aarde R., Whyte I. (2005). Landscape heterogeneity and the use of space by elephants in the Kruger National Park, South Africa. Afr. J. Ecol..

[45] Gough K.F., Kerley G.I.H. (2006). Demography and population dynamics in the elephants *Loxodonta africana *of Addo Elephant National Park, South Africa: Is there evidence of density dependent regulation?. Oryx.

[46] Spinage C. (1994). Elephants.

[47] Moss C.J. (2001). The demography of an African elephant (*Loxodonta africana*) population in Amboseli, Kenya. J. Zool..

